# Metagenomic Characterization of Indoor Dust Bacterial and Fungal Microbiota in Homes of Asthma and Non-asthma Patients Using Next Generation Sequencing

**DOI:** 10.3389/fmicb.2020.01671

**Published:** 2020-07-30

**Authors:** Jean-Pierre Gangneux, Mohamed Sassi, Pierre Lemire, Pierre Le Cann

**Affiliations:** Univ Rennes, CHU Rennes, Inserm, EHESP, Institut de Recherche en Santé, Environnement et Travail (Irset) – UMR_S 1085, Rennes, France

**Keywords:** microbiota, mycobiota, asthma, indoor environment, dust, next-generation sequencing, metagenomics, fungi

## Abstract

**Background:**

The exposure of house occupants to indoor air pollutants has increased in recent decades. Among microbiological contaminants, bacterial and fungal aerosols remain poorly studied and the debate on the impact of these aerosols on respiratory health is still open. This study aimed to assess the diversity of indoor microbial communities in relationship with the health of occupants.

**Methods:**

Measurements were taken from dwellings of 2 cohorts in Brittany (France), one with children without any pathology and the other with children and adults with asthma. Thirty dust samples were analyzed by next generation sequencing with a 16S and 18S targeted metagenomics approach. Analysis of sequencing data was performed using qiime 2, and univariate and multivariate statistical analysis using R software and phyloseq package.

**Results:**

A total of 2,637 prokaryotic (589 at genus level) and 2,153 eukaryotic taxa were identified (856 fungal taxa (39%) and 573 metazoa (26%)). The four main bacterial phyla were identified: Proteobacteria (53%), Firmicutes (27%), Actinobacteria (11%), Bacteroidetes (8%). Among Fungi, only 136 taxa were identified at genus level. Three main fungal phyla were identified: Ascomycota (84%), Basidiomycota (12%) and Mucoromycota (3%). No bacterial nor fungal phyla were significantly associated with asthma versus control group. A significant over representation in control group versus asthma was observed for Christensenellaceae family (*p*-value = 0.0015, adj. *p*-value = 0.033). Besides, a trend for over representation in control group was observed with Dermabacteraceae family (*p*-value = 0.0002, adj. *p*-value = 0.815).

**Conclusions:**

Our findings provide evidence that dust samples harbor a high diversity of human-associated bacteria and fungi. Molecular methods such as next generation sequencing are reliable tools for identifying and tracking the bacterial and fungal diversity in dust samples, a less easy strategy for the detection of eukaryotes at least using18S metagenomics approach. This study showed that the detection of some bacteria might be associated to indoor air of asthmatic patients. Regarding fungi, a higher number of samples and sequencing with more depth could allow reaching significant signatures.

## Introduction

Asthma is a common chronic inflammatory airway disease in children and adults from developed countries. In France, it affects more than 10% of children and 6% of adults (Delmas and Fuhrman, 2010).

Asthma is a very complex disease characterized by enhanced bronchial reactivity leading to airway obstruction and respiratory difficulties (Martinez and Vercelli, 2013). Exposure to environmental chemical or biological factors can lead to exacerbation of asthma and prevention measures are still needed in order to reduce the exposure to these agents (Beasley et al., 2015; Shaheen, 2019). The airway microbiome in asthma patients results from numerous factors. Environmental exposure, a complex relationship with the gastrointestinal microbiome, the development of immune function, and a predisposition to allergic sensitization and asthma are among the most referred factors (Huang and Boushey, 2015).

Indoor fungal exposure has been associated to the development of asthma (Chen et al., 2014). But, according to Ege et al. (2011), children who grow up in the environments with a wide range of microbial exposures, like farming environments, are more likely protected from childhood asthma and atopy than urban children. Feng et al. (2016) reported that parental allergic diseases, atopy, diet and early life exposures might explain the higher prevalence of asthma in the urban environment. Also, there is a complex interplay between genetic predisposition and environmental exposures, including microbes and allergens (Anderson and Jackson, 2017). Regarding exposure to molds, it is estimated that in France about 125,000 individuals suffer from severe asthma with fungal sensitization (SAFS) episodes (189 cases/100,000 adults per year) (Gangneux et al., 2016).

Among biological agents, bacteria have been less studied even if endotoxins produced by gram negative bacteria have been significantly correlated with wheezing in children (Horick et al., 2006). Moreover, many studies have suggested that exposure to lipopolysaccharide (LPS), in non-rural environments, is a risk factor for increased asthma prevalence and severity of disease (Thorne et al., 2005).

Culture-based studies have been in use for a long time for the detection of various microorganisms indoor (Rintala et al., 2008, 2012; Méheust et al., 2014). However, while culture isolation focuses on the presence of particular bacteria or fungi, next generation sequencing (NGS) studies unveil microbial communities comprising thousands of uncultured microbes. Recent advances in microbiological methods show promise in determining whether the microbes recovered during indoor sampling campaigns may in fact be causative agents as such, or only surrogates of an underlying harmful or beneficial exposure. Given the lack of knowledge on the specificities of the relevant exposures, assessments at this stage need to be kept broad and comprehensive (Mensah-Attipoe et al., 2017).

According to Singanayagam et al. (2017), who underlined the role of microbes in the exacerbation of asthma, the emergence of next generation molecular sequencing techniques to characterize the microbiota has facilitated renewed interest. They concluded that longitudinal studies that characterize the changes in lower respiratory tract microbiota from birth up to development of asthma are currently unavailable. Additionally, few studies have focused on the interactions between bacteria and the immune system in driving development of nonatopic asthma phenotypes.

Thus, despite the recognition of the importance of microbial exposure for human health, the precise role of microbes in the development and exacerbation of respiratory symptoms and allergies remains poorly understood.

The objective of our study was to use 16S and 18S rRNA sequencing approaches to characterize and identify indoor bacterial and eukaryotic communities that may be associated with the exacerbation of asthma. This pilot study compared two groups of asthmatic and non-asthmatic people’s dwellings.

## Materials and Methods

### Study Population and Home Visits

Thirty dust samples were collected from dwellings of 2 French cohorts and were analyzed by next-generation sequencing:

(i) 15 dwellings of asthma patients (ASTHMA) from The Ecenvir cohort, which aims to evaluate the clinical and the economic impact of Indoor Environment Counselor (IEC) on the symptoms of severe asthma. Patients answered a questionnaire during an interview with the IEC on dwellings characteristics (localization in cities or rural, apartment or house, …) and cleaning habits of patients. Asthma was graded with the GINA (Global Initiative for Asthma) scale and patients had three clinical check-ups.
(ii) 15 control dwellings of control patients (CONTROL) from the Pélagie AC cohort, which follows more than 3,500 pregnant women in Brittany from pregnancy to age of 20 of children. The Pélagie AC project aims to assess the effects on children respiratory health of chemicals and biological pollutants exposure. One of the check-up of Pélagie AC cohort occurs once the children are 6 years old. A subset of families without asthma answered a detailed questionnaire about their dwellings characteristics and life for the Pélagie AC study.

According to the French Public Health Law, protocols of this type are exempt from the requirement for formal informed consent. However, The INSERM and Rennes Ethical Committees approved the protocol, patients were informed and it was possible for them to refuse environmental samples for both cohorts:

– The Pélagie AC cohort, with dwellings being part of a larger study as stated in Dallongeville et al. (2015) (French Consulting Committee for the Treatment of Information in Medical Research (no. 09.485),
– The Ecenvir cohort, with dwellings being part of a larger study as approved by the Comité de Protection des Personnes de Rennes Ouest V (8 january 2013, N°ID RCB 2012-A01414-39).

### Dust Collection

Dust samples were collected by vacuuming the floor in the child’s bedroom using a Dustream Collector^TM^ (Indoor biotechnologies, United Kingdom) sampler-fitted vacuum cleaner (40 μm mesh nylon filter, domestic vacuum cleaner) as described by Dallongeville et al. (2015). Briefly, sampling was preferentially carried out on the carpets (when available) or on hard surface floors until filling at least 2/3 of the filter-sampler, in order to ensure collection of at least 10 mg of fine dust. Collected dust was sieved at 300 μm to discard debris, and 10 mg of fine dust were resuspended in 1 mL of PBS-Tween (0.05%) buffer, shaked at 800 rpm for 1 h and stored at −20°C until analyzed.

### DNA Extraction, Sequencing, and Analysis

Total DNA was extracted from samples using the method developed by Godon et al. (1997). A portion of the 16S rRNA was amplified using the barcoded, universal primer set (515WF/918WR) (Wang et al., 2009). DNA extraction was carried out according to the guanidium thiocyanate method. Positive extraction control is included. A portion of the 18S rRNA was amplified using universal 18S rRNA primer set (574WF/952WR) (Hadziavdic et al., 2014). PCR reactions were performed using AccuStart II PCR ToughMix kit and cleaned (HighPrep PCR beads, Mokascience). The amplification program consists of 28 PCR cycles with the following steps: 94°C for 3 min, followed by 28 cycles of 94°C for 30 s, 53°C for 40 s and 72°C for 1 min, followed by a final elongation step at 72°C for 5 min. PCR positive and negative controls are included. Pools were submitted for sequencing on Illumina MiSeq instrument at GeT-PlaGe (Auzeville, France). Sequencing read length was 250 bp. Sequences were processed using Mothur (version 1.33.1) according to MiSeq SOP pipeline (Schloss et al., 2009).

Barcodes, primers, and sequences showing homopolymers of more than 8 bp have been discarded. Sequences showing 100% homology were grouped in unique sequences, then in OTUs (operational taxonomic unit, based on 97% homology). Sequences were next assigned to match to a sequence in SILVA (version 123) and PR2 databases for prokaryotes and eukaryotes respectively to identify the genus level. We considered assigned sequences with relative abundance (RA) higher than 0.002 %.

### Sequencing Data Analysis

Analysis of sequencing data was performed using QIIME 2^1^, and univariate and multivariate statistical analysis using R software and phyloseq package.

## Results

### Comparison of Microbiota in the Environmental Samples

The sequencing of the 16S rRNA allowed the identification of 2,637 bacterial taxa, 589 at genus level with a predominance of Proteobacteria (53%), Firmicutes (27%) and Actinobacteria (11%). Regarding 18S, 2,153 eukaryotic taxa were identified: 856 fungal taxa (39%) and 573 metazoa (26%). Among Fungi, only 136 taxa were identified at genus level. The 16S and 18S sequencing data have been uploaded at EBI Metagenomics under the accession numbers PRJEB37043 and PRJEB37050 respectively.

### Bacteria

**FIGURE 1:**
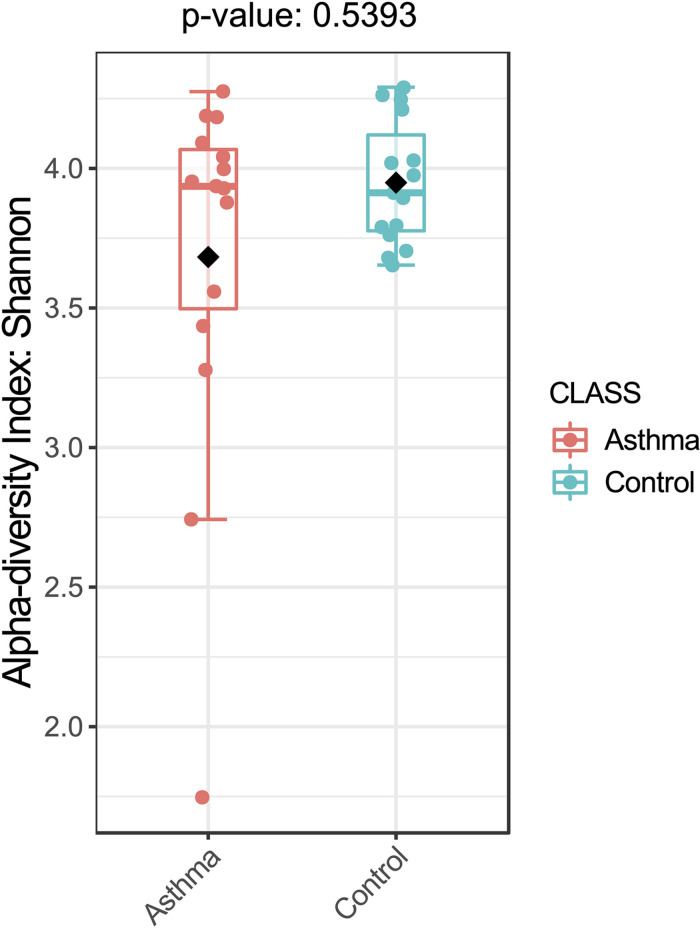
Bacterial alpha-diversity, measured by Shannon diversity Index is plotted for samples with asthma (red) and controls (blue). The line inside the box represents the median, while the whiskers represent the lowest and highest values within the 1.5 interquartile range (IQR). Outliers as well as individual sample values are shown as dots. Mann-Whitney statistical testing showed no significant difference in diversity between the two groups (pShannon = 0.5393).

**FIGURE 2:**
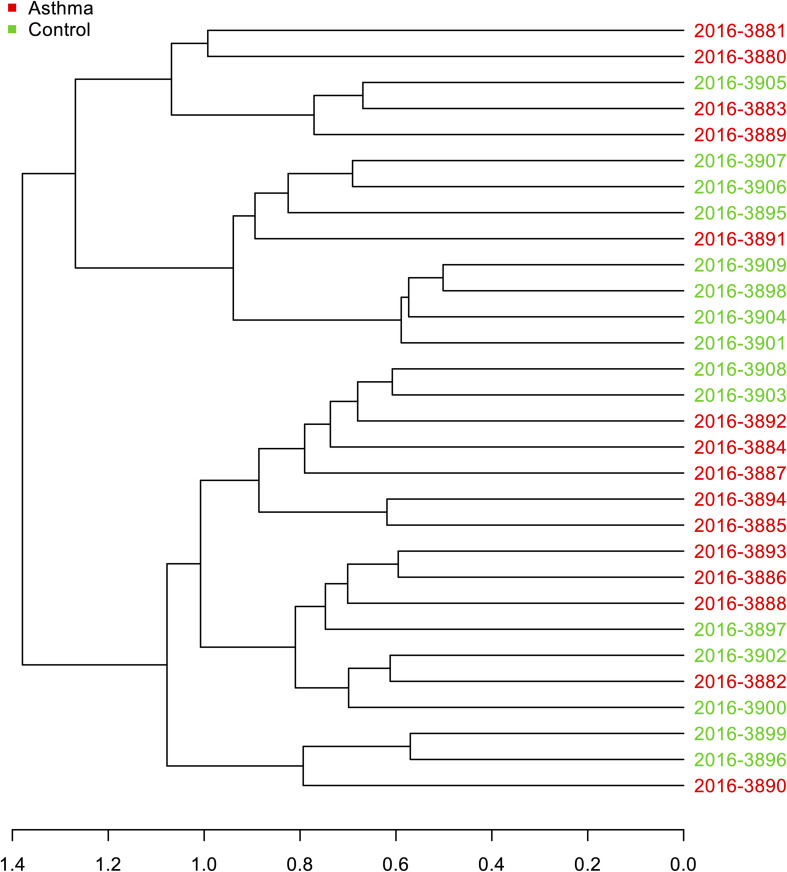
Hierarchical clustering analysis: Dendrogram of Bray-Curtis dissimilarity matrices between samples based on 16S data at feature level with UPGMA method.

**FIGURE 3:**
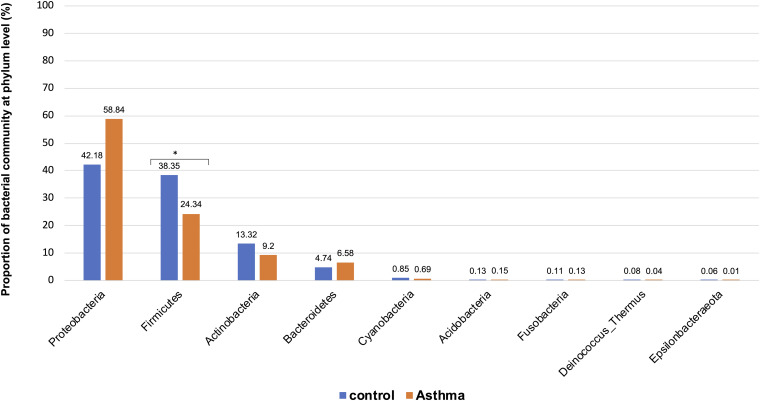
Proportion of bacterial community at phylum level in the control and asthma groups at the phylum level. *0.01 < *p* ≤ 0.05, based on Mann-Whitney *U*-test.

The Shannon index was used to evaluate the intra-group diversity in the environmental samples of the two groups; it was 3.68 ± 0.67 in the control group and 3.94 ± 0.22 in the asthma group, showing no significant difference in diversity between the two groups (Mann-Whitney statistical *p* = 0.5393) (Figure 1). The beta-diversity results using ANOSIM method indicated that the bacterial composition in the two groups was different as the global *R*-value was 0.109, with a *p*-value of 0.004. Hierarchical clustering analysis (Figure 2) indicated no distinct difference in the OTUs for the environmental samples of the two groups. There was no significant difference between the compositions of the bacterial communities in the environmental samples of the two groups, when analyzed at feature level (Supplementary Figure 1). When analyzed at the phylum level the abundance for the phylum Firmicutes was 38.35% in the control group, significantly higher than and 24.34% in the asthma group (24.34%, *p* = 0.02) (Table 1). Further, Proteobacteria, Actinobacteria and Bacteroides showed an abundance of 42.18, 13.32, and 4.74% in the control group and 58.84, 9.20, and 6.58% in the asthma group, respectively, with no significant difference between the compositions of the bacterial communities in the environmental samples of the two groups (Table 1, Figure 3 and Supplementary Figure 1).

**TABLE 1:** Abundances and percentages of the bacterial communities in the environmental samples of the control and asthma groups.

	Control		Asthma		Classical Univariate Statistical Comparisons			*Linear Discriminant Analysis (LDA) Effect Size (LEfSe)*					Deseq method			
					
Taxa	Abundance	Percentage	Abundance	Percentage	Pvalues	FDR	Statistics	Pvalues	FDR	Asthma	Control	LDAscore	log2FC	lfcSE	Pvalues	FDR
Proteobacteria	39305	42.18	67401	58.84	0.086611	0.27995	1.7759	0.071185	0.23728	5335600	4209300	−5.75	−0.54907	0.36825	0.13595	0.45318
Firmicutes	35733	38.35	27883	24.34	0.020982	0.10491	−2.4461	0.029436	0.14718	2639100	3859700	5.79	0.17782	0.2959	0.54788	0.93211
Actinobacteria	12415	13.32	10543	9.2	0.25007	0.50014	−1.1745	0.2902	0.72549	1052800	1317800	5.12	0.1867	0.28297	0.5094	0.93211
Bacteroidetes	4421	4.74	7542	6.58	0.11198	0.27995	1.641	0.60413	0.79302	850600	472570	−5.28	−0.90264	0.389	0.02032	0.1016
Cyanobacteria	790	0.85	791	0.69	0.93632	0.93632	0.08062	0.94825	0.94825	89040	86065	−3.17	−0.16357	1.1595	0.88782	0.98647
Acidobacteria	118	0.13	173	0.15	0.90185	0.93632	0.12445	0.71613	0.7957	13756	12537	−2.79	−0.54658	2.2049	0.80422	0.98647
Fusobacteria	101	0.11	154	0.13	0.67713	0.84641	0.42078	0.60465	0.79302	12344	7576.6	−3.38	0	0	1	1
Deinococcus_ Thermus	73	0.08	51	0.04	0.5005	0.73593	−0.68254	0.63442	0.79302	4478	8131.4	3.26	1.2533	2.1463	0.55927	0.93211
Epsilonbacteraeota	53	0.06	12	0.01	0.51515	0.73593	−0.65921	0.55026	0.79302	2283.1	6084	3.28	−0.85519	2.9528	0.77211	0.98647
Others	172	0.18	0	0	0.015326	0.10491	−2.5826	0.0075619	0.075619	0	20294	4.01	5.5643	2.1585	0.0099431	0.099431

### Fungi

**FIGURE 4:**
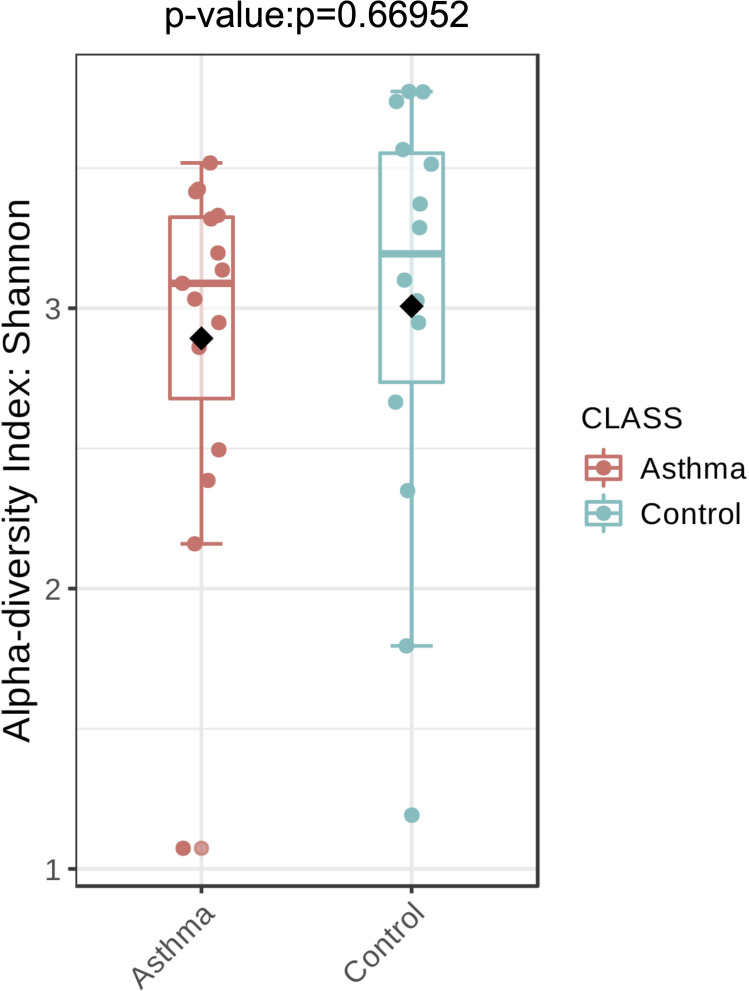
Fungal alpha-diversity, measured by Shannon diversity Index is plotted for samples with asthma (red) and controls (blue). The line inside the box represents the median, while the whiskers represent the lowest and highest values within the 1.5 interquartile range (IQR). Outliers as well as individual sample values are shown as dots. Mann-Whitney statistical testing showed no significant difference in diversity between the two groups (pShannon = 0.66952).

**FIGURE 5:**
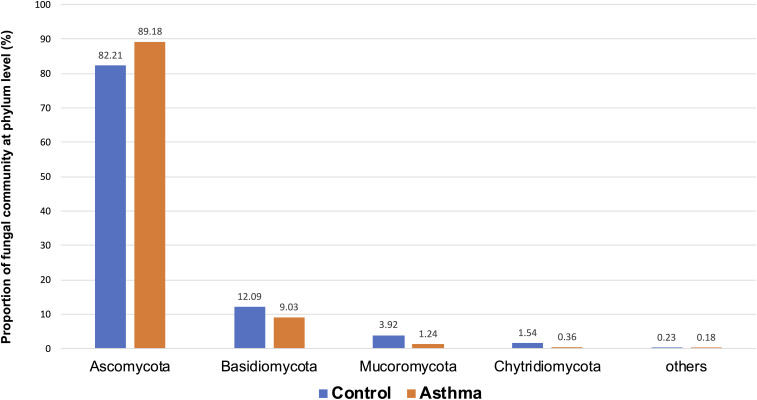
Proportion of fungal community at phylum level in the control and asthma groups at the phylum level. *0.01 < *p* ≤ 0.05, based on Mann-Whitney *U*-test.

The Shannon index was used to evaluate the intra-group diversity in the environmental samples of the two groups; it was 3.00 ± 0.77 in the control group and 2.89 ± 0.64 in the asthma group, showing no significant difference in diversity between the two groups (Mann-Whitney statistical *p* = 0.66952) (Figure 4). The beta-diversity results using ANOSIM method indicated that the fungal composition in the two groups was not different as the global *R*- value was -0.003, with a *p*-value of 0.859. There was no significant difference between the compositions of the fungal communities in the environmental samples of the two groups, when analyzed at feature level (Supplementary Figure 2) and phylum level (Table 2). For the phylum Ascomycota, the abundance was 82.21% in the control group and 89.18% in the asthma group. Furthermore, Basidiomycota and Mucoromycota showed an abundance of 12.09 and 3.92% in the control group and 9.03 and 1.24% in the asthma group, respectively (Figure 5).

**TABLE 2:** Abundances and percentages of the fungal communities in the environmental samples of the control and asthma groups.

	Control		Asthma		Classical Univariate Statistical Comparisons			*Linear Discriminant Analysis (LDA) Effect Size (LEfSe)*					Deseq method			
					
Taxa	Abundance	Percentage	Abundance	Percentage	Pvalues	FDR	Statistics	Pvalues	FDR	Asthma	Control	LDAscore	log2FC	lfcSE	Pvalues	FDR
Ascomycota	136492	82.21	169747	89.18	0.11207	0.31014	142	0.10635	0.29345	8744400	8042200	−5.55	−0.39748	0.40435	0.3256	0.51503
Basidiomycota	20078	12.09	17194	9.03	0.18608	0.31014	74	0.17607	0.29345	996370	1388100	5.29	0.22401	0.22313	0.31541	0.51503
Mucoromycota	6515	3.92	2357	1.24	0.91251	0.91251	108	0.89511	0.89511	201920	358010	4.89	−1.2848	0.99581	0.19698	0.51503
Chytridiomycota	2564	1.54	691	0.36	0.16554	0.31014	75	0.15849	0.29345	33275	185200	4.88	0.99249	1.4868	0.50444	0.51503
Others	376	0.23	352	0.18	0.33805	0.42257	123.5	0.32481	0.40601	24060	26473	3.08	−1.2974	1.9928	0.51503	0.51503

### Differential Abundance Analysis

**FIGURE 6:**
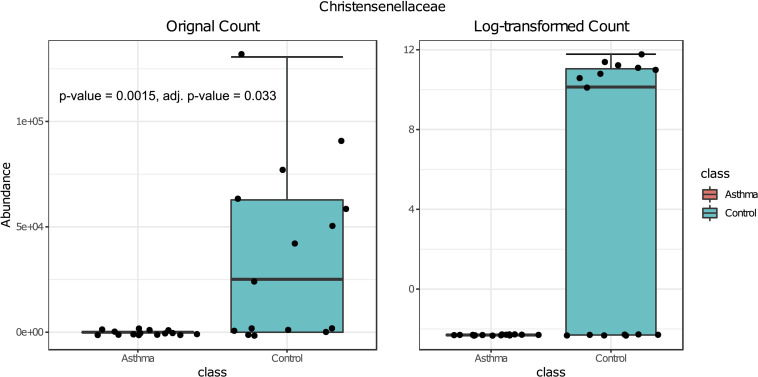
Box plot of differential abundance of Christensenellaceae family in the control and asthma groups using Classical Univariate Statistical Comparisons method based on Mann-Whitney *U*-test.

**FIGURE 7:**
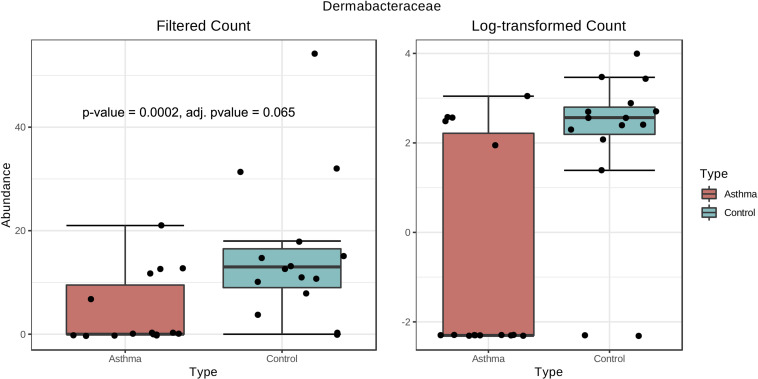
Box plot of differential abundance of Dermabacteraceae family in the control and asthma groups using Classical Univariate Statistical Comparisons method based on Mann-Whitney *U*-test.

Bacterial and fungal taxon-levels (OTU) differences in relative abundance were examined between the asthma and control groups. A significant over representation of specific bacteria was observed in control patients’ group for Christensenellaceae family (*p*-value = 0.0015, adj. *p*-value = 0.033) (Figure 6) and only a trend for Dermabacteraceae family (*p*-value = 0.0002, adj. *p*-value = 0.065) (Figure 7).

## Discussion

Health effects of environmental microbes are still debated and not completely understood. A first approach is to examine relationships between genes, microbes in airways and gut, and the environment in asthma causes. This is a complex, dynamic and very heterogeneous process still unresolved (Huang and Boushey, 2015). Besides, healthy environments may have impact on the exacerbation of asthma and the usefulness of allergen avoidance on asthma control have shown various evidence of their efficacy on the clinical improvement of patients (Le Cann et al., 2017; Gangneux et al., 2020). In this work, our aim was to compare microbial communities in asthma patients dwellings compared to non-asthma control homes, and to characterize and identify indoor bacterial and eukaryotic communities that may be associated with the exacerbation of asthma. In the literature, exposure to low fungal and bacterial richness in house dust is associated with an increased risk of asthma development (Ege et al., 2011; Dannemiller et al., 2013). Air, dust and surface sampling strategies to detect bacteria and fungi have alternately advantages and limits, as well as the different methods of detection, identification and quantification (Méheust et al., 2014; Gangneux et al., 2019). Because our objective for this work was to use NGS, we decided to sample dust by vacuuming the floor rather than aerosols in order to gain in sensitivity. Another valuable technical option described in the literature relies on the use of electrostatic dust cloths for further molecular, immunological or cultural analysis (Cox et al., 2017; Kristono et al., 2019).

In our work, the alpha- and beta-diversity results indicated that the bacterial and fungal composition in the two groups were not different, without significant difference when analyzed at the phylum level. On a global point of view, our results are in accordance to others as the main phylum detected in both group is Proteobacteria, then Firmicutes, Actinobacteria, Bacteroidetes (Hewitt et al., 2012), with Firmicutes over represented in the control group compared to asthma dwellings. Besides, there was a trend of more Proteobacteria detected in asthma population as previously described (Ciaccio et al., 2015). This was associated with a higher abundance of Proteobacteria in airways microbiota of asthmatic people (Hilty et al., 2010). However, it is very difficult to compare the studies because usually the populations studied are different (sometimes urban, sometimes rural, etc…). At the family level, a signature in differential abundance was observed with significantly more Christensenellaceae detected in control dwellings than in asthma homes. Regarding other genus and family, no statistical difference was observed even if much Dermatobacteriacae were also detected in control dwellings.

Some limitations of this work can be identified. A lack of statistical power is possible, linked to the limited number of dwellings sampled. However, this study opens new perspectives with interesting trends that may drive further studies on the topic. Also, it is possible that we have underestimated the fungal burden and diversity in dust samples because of a potential loss of fungal spores as shown for aerosols (Mbareche et al., 2019). The interest of concentrating particles before DNA extraction needs to be investigated on dust samples as it has been studied for air samples. Finally, we intentionally decided to amplify and sequence rRNA of 18S gene in order to detect fungi but also eukaryotes cells that may be involved in asthma sensitization such as cockroach, house dust mites or pets. However, we were very disappointed by the low resolution for species identification of non fungal eukaryotes and were not able to analyze them. Thus, if looking only to fungi, the question of using ITS1-2 or 28S rRNA targets could have been asked, while advantages and limits of each target and even primer sets are still debated (Hanson et al., 2016; De Filippis et al., 2017; Frau et al., 2019). Another option is to use shotgun sequencing for taxon identification and abundance assessment for both prokaryotic and eukaryotic sequences. New tools such as NGS should be more standardized from one study to another and include processing steps of quality controls in order to avoid low-quality reads and sequences and to optimize abundance thresholds and profiles. However, deep sequencing really opens new perspectives on the comprehension of the microbial exposome and its impact on airway microbiome.

## Data Availability Statement

The datasets generated for this study are available on request to the corresponding author.

## Ethics Statement

According to the French Public Health Law, protocols of this type are exempt from the requirement for formal informed consent. However, they were informed and it was possible to refuse environmental samples for both cohorts.

## Author Contributions

All authors listed have made a substantial, direct and intellectual contribution to the work, and approved it for publication.

## Conflict of Interest

The authors declare that the research was conducted in the absence of any commercial or financial relationships that could be construed as a potential conflict of interest.

## Footnotes

1 https://qiime2.org

## Supplementary Material

FIGURE S1 Heatmap based on 16S analysis. The Euclidean dissimilarity matrix and Ward’s hierarchical clustering algorithms were applied in this analysis. Rows indicate feature level (OTUs) and columns indicate the different samples.

FIGURE S2 Heatmap based on 18S analysis. The Euclidean dissimilarity matrix and Ward’s hierarchical clustering algorithms were applied in this analysis. Rows indicate feature level (OTUs) and columns indicate the different samples.
